# Role of Cluster of Differentiation 163 in Diabetes-Periodontitis Interplay

**DOI:** 10.7759/cureus.8523

**Published:** 2020-06-09

**Authors:** Daliah Ruth, Jaideep Mahendra, Anilkumar Kumar, Ambalavanan Namasivayam, Little Mahendra, Nalini Devarajan

**Affiliations:** 1 Periodontics, Meenakshi Academy of Higher Education and Research, Meenakshi Ammal Dental College and Hospital, Chennai, IND; 2 Periodontics, Meenakshi Ammal Dental College and Hospital, Chennai, IND; 3 Periodontics, Maktoum Bin Hamdan Dental University College, Dubai, ARE; 4 Research, Central Research Laboratory, Meenakshi Academy of Higher Education and Research, Meenakshi Ammal Dental College and Hospital, Chennai, IND

**Keywords:** cd163, chronic periodontitis, diabetes, biomarker, protein, wound healing

## Abstract

Background

The aim of the present study was to assess and quantify cluster of differentiation 163 (CD163) protein levels and CD163 messenger RNA (mRNA) gene expression in subgingival plaque samples of generalized chronic periodontitis subjects with and without type II diabetes mellitus (DM).

Materials and methods

Eighty chronic periodontitis subjects were selected and divided into 40 systemically healthy, generalized chronic periodontitis subjects (Group I) and 40 generalized chronic periodontitis subjects diagnosed with type II diabetes mellitus (Group II). Age, body mass index (BMI), income, plaque index (PI), bleeding on probing (BOP), probing pocket depth (PPD), and clinical attachment level (CAL) were recorded. CD163 protein and gene expressions were quantified and compared between the groups.

Results

The mean age, BMI, income, PI, BOP %, and CD163 protein and gene expression were higher in Group II (p< 0.05) as compared to Group I. In Group I, CD163 protein levels showed a negative correlation with respect to BMI and PI, and this was statistically significant. In Group II, all the periodontal parameters showed a positive correlation with CD163 protein levels. Overall, PI and BOP % were significantly correlated with CD163 protein levels. Both CD163 protein and gene expression showed a negative correlation with each other (p= 0.001).

Conclusion

The elevated protein levels of CD163 in the subgingival plaque samples of generalized chronic periodontitis individuals with type II diabetes mellitus signify the involvement of CD163 in the pathogenesis of both periodontitis and diabetes mellitus. CD163 can play a challenging role as a diagnostic, as well as a prognostic biomarker, in both these inflammatory diseases.

## Introduction

Periodontitis is a microbial-induced chronic inflammatory disease of supporting periodontal structures resulting in progressive destruction of the periodontium with an increase in probing pocket depth, recession, or both, leading to tooth loss. Periodontal disease results from a complex interplay between subgingival biofilm and the host immune-inflammatory events. It is important to understand the cause and pathological process of periodontal diseases and their chronic inflammatory nature to reveal the possible way through which it may activate several infectious events in the body [1].

Diabetes mellitus (DM) is a group of metabolic disorders characterized by decreased insulin secretion, insulin resistance, or both, causing a hyperglycemic state. The elevated inflammatory state in diabetes contributes to both microvascular and macrovascular complications and it is clear that hyperglycemia can result in the activation of pathways that increase inflammation, oxidative stress, and apoptosis. The primary complications of DM are heart disease, stroke, hypertension, kidney disease, diseases of nerves, and oral infections. Secondary complications of uncontrolled diabetes mellitus include nephropathy, retinopathy with possible blindness, neuropathy, and delayed tissue healing [2].

In the 1990s, it has been reported that periodontitis is considered the sixth complication of type II diabetes mellitus (T2DM) [3]. In diabetic individuals, oral microflora concentrations are increased due to the high glucose level in saliva and gingival crevicular fluid [4]. Diabetic patients have higher periopathogenic bacteria, which results in a state of an exaggerated immune response, leading to more rapid and severe periodontal destruction.

In chronic periodontitis (CP), the gram-negative periodontal pathogens release virulence products and reactive oxygen species (ROS), which induce an inflammatory response in the host by increasing oxidative stress in tissue, thereby facilitating insulin resistance and severe tissue destruction [5-6]. It also results in increased production of advanced glycation end products (AGEs). All these structural and functional aspects are modulated by proteins and act as an important component in the metabolic pathways of cells. Although the presence of bacterial components, associated risk factors, and the host immune response plays a pivotal role in the disease state, the bacterial components initiate these processes, which result in tissue destruction [5].

Proteins are the structural and functional units of many activities in the body. They can play an important role as an effective biomarker and can be objectively measured and evaluated as indicators of pathogenic processes or biological responses to the treatment modalities. Cluster of differentiation 163 (CD163) is a type I transmembrane protein of 130kD molecular weight belonging to group B of the scavenger receptor cysteine-rich (SRCR) superfamily. Recently, CD163 has been proposed as a specific marker of monocytes/macrophages cell populations exhibiting strong inflammatory properties. It also transduces signals upon binding of its ligands that lead to the release of inflammatory mediators such as interleukin-10 (IL-10) [7].

In recent years, the CD163 receptor has also been reported to bind the tumor necrosis factor-α (TNF-α)-like weak inducer of the apoptosis (TWEAK) protein, pathogenic bacteria, and virus. The levels of sCD163 are elevated in bacteremia and in community-acquired severe septic conditions [8]. The macrophage is the cell responsible for the release of CD163 and is an important immune cell in the pathogenesis of periodontitis [9].

Insulin resistance is associated with an increased concentration of inflammatory markers such as C-reactive protein (CRP). In addition, the adipose tissues are infiltrated by macrophages that secrete pro-inflammatory cytokines such as TNF-α and IL-6 [10]. These cytokines promote insulin resistance and plasma sCD163, which is regarded as a longer duration circulating marker of TNF-α. Hence, the tissue and serum concentrations of sCD163 are increased in type II diabetic patients with obesity [11]. 

Though in the past, literature has suggested the role of CD163 in periodontitis, however, its role in patients with diabetes mellitus and periodontal inflammation has not been explored so far. To the best of our knowledge, this is the first study in the South Indian population that attempts to quantify CD163 protein levels and gene expression in chronic periodontitis patients with type II diabetes mellitus. In the present study, we aimed to assess the demographic variables such as age, BMI, monthly income, and periodontal parameters: plaque index (PI), bleeding on probing (BOP), probing pocket depth (PPD), and clinical attachment level (CAL), and correlate CD163 protein levels and gene expression with the amount of periodontal destruction in subgingival plaque samples of generalized chronic periodontitis patients with and without type II diabetes mellitus.

## Materials and methods

Study design

**Figure 1:**
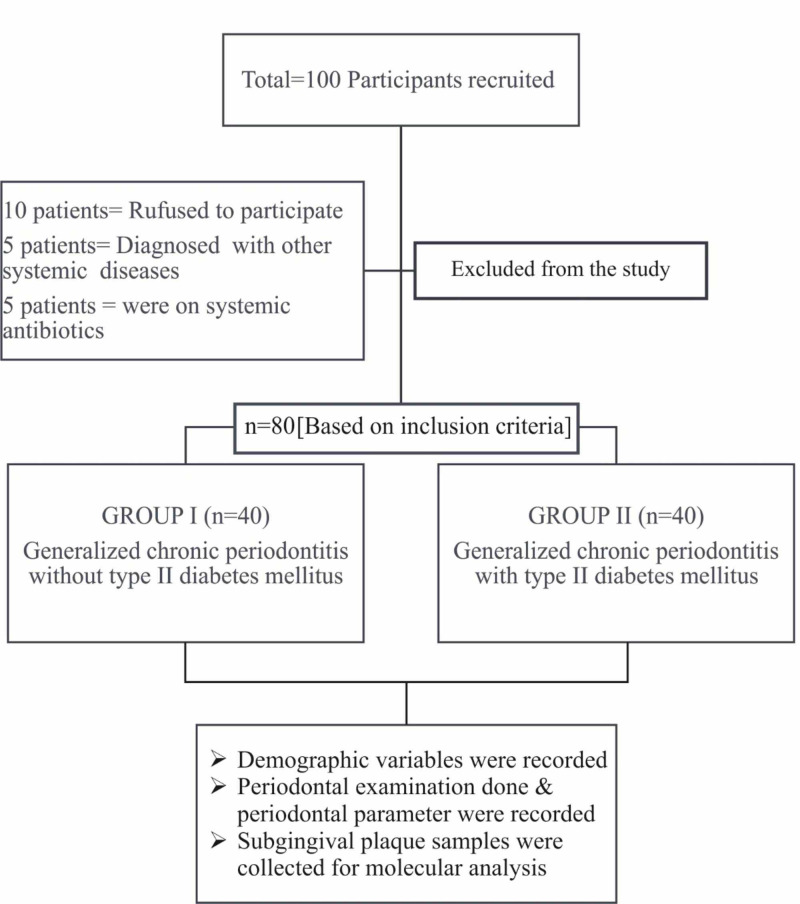
Flowchart of Study design

The study was conducted from August 2018 to September 2019 in Chennai, Tamil Nadu, India. A total of 100 subjects was recruited from the outpatient department of periodontics, faculty of dentistry, Meenakshi Academy of Higher Education and Research. Out of 100 patients, 10 refused to participate in the study, five patients were diagnosed with other systemic diseases and five subjects were on systemic antibiotics and hence were excluded from the investigation. Finally, 80 subjects with generalized chronic periodontitis were selected and were divided into two groups - Group I: 40 generalized chronic periodontitis subjects without type II diabetes mellitus and Group II: 40 generalized chronic periodontitis subjects diagnosed with type II diabetes mellitus (Figure 1). Inclusion criteria for both the groups included: patients within the age group of 30-65 years (both male and female) who were willing to participate in the study, the presence of ≥ 10 natural teeth, subjects having 30% or more sites with clinical attachment loss (CAL) ≥ 5 mm. For group II, diabetic subjects with glycated hemoglobin (HbA1C) 7%-8% were included [12]. The subjects with systemic conditions, such as respiratory diseases, renal diseases, liver diseases, rheumatoid arthritis, allergy, advanced malignancies, and human immunodeficiency virus (HIV) infection, patients on drugs such as corticosteroids, antibiotics, aspirin, within three months of investigation, current smokers, individuals who quit smoking less than six months ago, who had undergone periodontal therapy within the previous six months and pregnant women (which may alter the oral flora) were excluded from the present investigation. The study was approved by the human subjects ethics board of MAHER - Deemed to be University, Chennai, India (“Institutional Review Board”, Protocol No: MADC/IRB-XV/2017/302) and was conducted in accordance with the Helsinki Declaration of 1975, as revised in 2013. The study was also registered with ClinicalTrials.gov (ID: NCT03727035). The subjects were explained about the study and written informed consent was obtained from those who agreed to voluntarily participate in this study.

Demographic data

The power of the study was calculated and it was found to be more than 95%, with a sample size of 40 subjects in each group.

Demographic information was collected and recorded from the two groups: (a) Age (range, 35‐65 years); (b) BMI (kg/m^2^); and (c) Socioeconomic status of the patients based on monthly income. All participants were from the urban and suburban areas of the South Indian population.

Periodontal screening and examination

Periodontal examination was performed by two proficient and calibrated investigators (J.M. and D.R.) blinded to the study groups. Calibrations were completed before the start of the study in the outpatient department of periodontology, Meenakshi Ammal Dental College and Hospital, Chennai. Periodontal parameters were recorded with a Williams Probe (Hu‐Friedy, Chicago, IL). These included: (a) PI; (b) BOP; (c) PPD; and (d) CAL [13-14]. The bleeding on probing percentage was evaluated as bleeding sites per tooth (mesiofacial, facial, distofacial, and lingual) [13]. PPD and CAL were also assessed in six sites per tooth (mesiofacial, facial, distofacial, mesiolingual, lingual, and distolingual) and the measurement was recorded to the nearest millimeter. Participating subjects were diagnosed with periodontitis if at least 30% or more sites with probing pocket depth (PPD) and clinical attachment loss (CAL) ≥ 5 mm [14].

Collection of subgingival plaque samples

In both the groups, subgingival plaque samples were obtained with Gracey curettes (Hu‐Friedy, Chicago, IL) from the deepest periodontal sites of CP patients with and without type II DM. The plaque samples were then mixed with RNAlater solution (Qiagen, Hilden, Germany) to prevent the degradation of transcriptomes and stored at -80°C for further analysis.

Molecular analysis

CD163 Protein Analysis by Enzyme-Linked Immuno-Sorbent Assay (ELISA)

The protein concentration of CD163 in both groups was analyzed by ELISA (Bioassay Technologies Laboratory, Shanghai, China). Kits were used according to the manufacturer’s instructions. All the standards solutions and samples were prepared and brought to room temperature before use. To standardize the procedure, the standards of various concentrations were added to the blank wells. To these, 50 µl of the sample diluent was added The assay was performed at room temperature in the 96-well plates. Forty μl of the sample was added, followed by 10 µl anti-CD163 antibody and 50 µl streptavidin-HRP. The plate was covered with a sealer and incubated for 60 minutes at 37^0^C. The sealer was removed and the plate was washed five times with wash buffer. This was followed by adding 50 µl substrate A & B solutions to each well, and the plate was covered with a new sealer for 10 minutes at 37^0^C in the dark. To end the reaction, a 50 µl stop solution was added, which turned the blue color into yellow immediately. After adding the stop solution, the plate was kept in the plate reader within 10 minutes. Optical densities were measured at 450 nm and plotted against a standard curve to determine protein concentrations.

CD163 mRNA Expression (RT-PCR)

The subgingival plaque samples were subjected to a real‐time polymerase chain reaction (RT‐PCR) for expression and quantification of CD163. Total ribonucleic acid (RNA) was isolated from the samples using an RNA isolation kit (RNA iso plus) according to the manufacturer's instructions. This RNA sample was quantified by a spectrometer (Nanodrop One; Thermo Fisher Scientific; Waltham, Massachusetts) by measuring absorbance (A) at 260/280 nm and 230/280 nm. Complementary deoxyribonucleic acid (cDNA) synthesis and reverse transcription-polymerase chain reaction (RT‐PCR) analysis was performed using a kit (Thermo Scientific™ RevertAid™; Thermo Fisher Scientific), Assay Kit (RevertAid Reverse Transcriptase; Thermo Fisher Scientific), and Universal Master Mix solution (RiboLock™ RNase Inhibitor, Thermo Fisher Scientific). Real-time PCR (RT-PCR) amplification was performed in triplicate following the instructions of the Takara kit (TB GreenTMPremix Ex Taq TMII PCR Kit; Takara Bio Inc., Shiga, Japan), which included three-step cycling of denaturation for 5 min at 95° C, annealing at 55°C for 30 s, and extension for 30 s at 72°C for 40 cycles.

The forward and reverse primers (Takara#RR820A) sequences for CD163 were 5’TGGTGAGAAGGGTGAGAA-3’, 5’-AGATCTTGGTAAAGCGAATG-3’, and primers of GAPDH (housekeeping gene) were 5’AGCCACATCGCTCAGACAC-3’,5’-GCCCAATACGACCAAATCC-3’, respectively. Results were analyzed using the CFX96 Touch Real-Time system (BioRad system; Bio-Rad Laboratories, Inc., Hercules, California). Finally, the CD163 mRNA expressions were calculated using the comparative computed tomography (CT) method. Statistical analysis was done using statistical software (SPSS, version 20.1, IBM Corp., Armonk, NY). Mean and SD for all the parameters were estimated for both the groups and the level of significance was determined. To find the significant difference of all the variables between both groups, multivariate analysis and the unpaired t-test were used. Pearson's correlation test was used to evaluate the relationship between variables and CD163 levels. In the present study, p-value <.05 was considered statistically significant.

## Results

Comparison of variables and CD163 levels between the groups

The mean and standard deviation (SD) for demographic variables, periodontal parameters, CD163 protein levels, and CD163 mRNA gene expression was compared between the groups. Age, BMI, monthly income, PI, BOP(%), CD163 protein levels, and CD163 mRNA gene expression showed statistical significance at p<.05 (Table 1).

**Table 1:** Comparison of all the variables between Group I and Group II NS: Not significant; *: Statistically significant; BMI: body mass index; PI: plaque index; BOP: bleeding on probing; PPD: probing pocket depth; CAL: clinical attachment level

Variables	Mean ± Standard Deviation		t-value	p-value
	Group I (Control Group)	Group II (Test Group)		
Age (years)	43.65 ± 8.92	49.12 ± 6.26	3.176	0.010*
BMI	26.84 ± 2.71	30.49 ± 3.87	4.886	0.025*
Income per month (Rs)	18352.90 ± 15456.87	18243.37 ± 15923	0.232	0.052*
PI	1.90 ± 0.42	2.36 ± 0.30	5.653	0.002*
BOP (%)	97 ± 14	81 ± 0.1	0.605	0.047*
PPD (mm)	5.02 ± 4.58	4.66 ± 0.47	2.810	0.087^NS^
CAL (mm)	4.47 ± 0.72	5.88 ± 6.84	2.416	0.140^NS^
Quantification of CD163 protein (ng/l)	1.81 ± 0.63	6.61 ± 0.82	29.267	0.0001*
Quantification of CD163 mRNA gene (fold)	22.87 ± 0.81	18.36 ± 5.43	6.921	0.0001*

Correlation of variables with CD163 levels in group I and group II

On correlating, all the variables with CD163 protein and gene expression in the CP group (Group I), it was found that BMI and plaque index showed a significant correlation (p<.01) (Table 2). When the demographic variables and periodontal parameters in CP+DM (group II) were correlated with CD163 protein levels, PI, BOP (%), PPD, and CAL showed a positive correlation (p<.01) (Table 3).

**Table 2:** Correlation of all the demographic variables and periodontal parameters with protein quantification and mRNA expression of CD163 in Group I (control group) NS: Not significant; *: Statistically significant; mRNA: messenger ribonucleic acid

Variables		Quantification of CD163 protein (ng/l)	Quantification of CD163 mRNA gene expression (Fold)
Age (years)	Correlation	-0.101	-0.059
	p-value	0.53^NS^	0.58^NS^
BMI	Correlation	-0.354	-0.037
	p-value	0.02*	0.82^NS^
Income per.month (Rs)	Correlation	0.429	0.214
	p-value	0.32^NS^	0.83^NS^
Plaque index	Correlation	-0.302	0.008
	p-value	0.05*	0.96^NS^
Bleeding on probing (%)	Correlation	0.302	-0.286
	p-value	0.58^NS^	0.07^NS^
Probing pocket depth (mm)	Correlation	-0.229	-0.161
	p-value	0.15^NS^	0.32^NS^
Clinical attachment level (mm)	Correlation	-0.204	-0.104
	p-value	0.20^NS^	0.52^NS^

**Table 3:** Correlation of all the demographic variables and periodontal parameters with protein quantification and gene expression of CD163 within Group II (Test group) NS: Not significant; *: Statistically significant

		Quantification of CD163 protein (ng/l)	Quantification of CD163 mRNA gene expression (Fold)
Age (years)	Correlation	-0.034	0.062
	p-value	0.76^NS^	0.58^NS^
BMI	Correlation	0.026	0.126
	p-value	0.41^NS^	0.26^NS^
Income per. month (Rs)	Correlation	0.429	0.214
	p-value	0.32^NS^	0.83^NS^
Plaque index	Correlation	0.578	-0.701
	p-value	0.001*	0.001*
Bleeding on probing (%)	Correlation	0.566	-0.717
	p-value	0.001*	0.001*
Probing pocket depth (mm)	Correlation	0.608	-0.738
	p-value	0.001*	0.001*
Clinical attachment level (mm)	Correlation	0.606	-0.732
	p-value	0.001*	0.001*

## Discussion

The influence of chronic systemic conditions on the periodontium has been extensively studied. The pathogenesis of type II diabetes alters host immunity through bacteremia-stimulating periodontal destruction. Earlier researches by Collin et al. and Grossi et al. have elaborated on the influence of uncontrolled hyperglycemia on the periodontium by diminishing the gingival fibroblast and the synthesis of collagen, thereby enhancing the crevicular fluid collagenolytic activity resulting in periodontal destruction [15]. Ohlrich et al. also explained the bidirectional link between diabetes and periodontitis in subgingival plaque samples [16].

Although proteins are the keystone players that are responsible for the functions of cells, their expression, localization, and involvement differ in various diseases. Recently, sCD163 has been identified as a biomarker for the diagnosis, prognosis, and monitoring of disease progression. Reports by Detzen et al. have revealed the association of CD163 in saliva with periodontitis subjects [17]. Several studies have already proven that the subgingival biofilm is a primary reservoir of pathognomonic bacteria and cytotoxic agents in established periodontitis. In the present study, we aimed to quantify CD163 protein levels and its expression in the subgingival plaque samples of generalized chronic periodontitis patients with and without type II diabetes mellitus and correlate it with the amount of periodontal destruction.

In our study, demographic variables, such as age, BMI, and monthly income, were higher in CP+DM (Group II) as compared to CP alone (Group I). This was consistent with the study done by Bennet et al. in Pima Indians [18]. He stated that higher aged individuals showed a greater incidence of periodontal disease and the rate of progression of periodontal disease in subjects with diabetes was 2.6 times greater than those without diabetes. Harald Loe reported that above 35 years of age, there is a strong prediction of developing periodontal disease, which also attributes to the development of diabetes [19]. In our study, BMI was found to be higher in the CP+DM group (p=0.025) than CP. It was in accordance with Suvan et al., who in their meta‐analyses indicated a stronger association between obesity and periodontitis [20]. According to him, the odds ratio of developing periodontitis in obese individuals is 1.8 times greater than that in those with normal BMI. Therefore, with an increase in BMI, there is an increased risk for periodontitis. Similarly, a report by Snophia S and Mahendra J explained that the adipose tissue functions as the endocrine organ, which secretes numerous factors referred to as adipocytokines and causes disease by dysregulated immune responses [21]. These adipocytokines activate monocytes, thereby increasing the inflammatory cytokines, which eventually results in the initiation of periodontal disease and mediates the link between insulin resistance and periodontal disease. In our study, we also observed that the CP+DM group, which had higher BMI, also showed higher CD163 levels of protein and gene expression. This was in accordance with Sorensen et al., who studied that sCD163 concentration was significantly greater in obese type II diabetes mellitus individuals as compared with normal-weight individuals [22]. In our study, the socioeconomic status was comparatively lower in the CP+DM group and this was similar to Larranaga et al. who linked the individuals with poor education and low socioeconomic status with a high prevalence of type II diabetes mellitus that subsequently resulted in compromised periodontal conditions [23]. Thus the statistical significance of the above variables in CP+DM explains the link that prevails between the inflammatory pathways in aged individuals with higher BMI and low socioeconomic status.

In our study, PI and BOP (%) were significantly higher in the CP+DM group. This was consistent with Emrich et al. and Pham et al. who stated that the plaque index and BOP were higher among patients with periodontitis and diabetes mellitus when compared to healthy individuals [24-25]. The possible etiology could be due to the host-immune interaction that results in an inflammatory response that ends up with periodontal destruction. Silva and co-workers demonstrated the interaction of the host's immune system with the bacteria in dental plaque, consisting of microbial colonies resulting in the thickening of the gingival epithelium, elongated dermal papilla, and disorganized collagen alignment in connective tissue with a prominent inflammatory cell infiltrate in diabetic subjects [26]. Furthermore, the diabetic condition can prolong the period of periodontal breakdown, which may delay mitogenesis. Thus, this explains the relationship between a consistent amount of plaque accumulation and periodontal destruction, thereby endorsing the increased risk of developing severe periodontitis in diabetes mellitus subjects.

CAL and PPD were more or less similar in both groups. However, this was in contrast with the study done by Farzeen et al. who found CAL and PPD to be higher in uncontrolled diabetic patients and revealed that uncontrolled diabetic patients tend to have more periodontal inflammation and destruction when compared to controlled diabetic patients [27]. The subjects chosen in our study were controlled diabetics with HbA1c (7%-8%) [12]. The insignificant result between the two groups in our study could be due to the fact that both groups had chronic periodontitis with similar pocket depth and CAL. The periodontal parameters, such as PPD and CAL, show only past events and not the current disease state and inflammation. Hence, CAL did not show the current disease progression in controlled diabetic subjects selected in this study.

In the present study, protein quantification and the mRNA gene expression of CD163 was detected and expressed in subgingival plaque samples of both the groups. CD163 expression in the CP+DM group was found to be higher than in the CP group, indicating significantly elevated expression. This was consistent with the study done by Min et al., who found increased circulating sCD163 levels in diabetic subjects as compared to controls [28]. Similarly, Parkner et al. stated that sCD163 is a strong and independent predictor of insulin resistance and the levels increased significantly with deteriorating glycemic control [22]. Allam et al. and Sima and Glogauer identified that CD163 plays an important role in the pathophysiology of periodontitis, which is directly related to the IL-17 producing T-cell, which is a potent pro-inflammatory cytokine that stimulates polymorphonuclear neutrophil (PMN) recruitment and activation. It also produces IL-23 in response to toll-like receptor-2 (TLR-2) and toll-like receptor-4 (TLR-4) activation by Porphyromonas gingivalis [29].

In this study, when we correlated the protein and gene expression of CD163 with the demographic variables and periodontal parameters in both groups, we found that though in CP (Group I), CD163 levels did not show a positive correlation with demographic and periodontal parameters (Table 2), however, in the CP+DM (Group II), it showed a significant positive correlation with PI (p-value 0.001), BOP (p-value 0.001), PPD (p-value 0.001), and CAL (p-value 0.001) (Table 3). This was in accordance with the Detzen et al. study, where they reported that CD163 expression in saliva and serum samples of periodontitis patients was highly correlated with periodontal parameters suggesting the relationship between CD163 levels and periodontal destruction [17]. Therefore, an increased level of CD163 in our study provides a better understanding of its role in the pathology of periodontal destruction in type II diabetes mellitus.

To the best of our knowledge, this is the first study to quantify protein CD163 in the subgingival plaque samples of chronic periodontitis subjects with and without type II diabetes mellitus. Earlier studies have analyzed the above biomarker in serum, saliva, and tissue; however, analyzing the CD163 protein from subgingival plaque has not been attempted. Within the limitation of this study, CD163 protein quantification has the insight to assess the progression of periodontitis and its impact on metabolic diseases, such as diabetes mellitus, thereby making them potent biomarkers for both these inflammatory conditions.

In the future, the CD163 protein can aid as a signaling molecule in the earlier detection of periodontal disease and diabetes mellitus. This may become a target point for the development of therapeutic drugs in both diseases. Furthermore, this study enlightens the link that prevails between the molecular aspects of diabetes and chronic periodontitis, which could provide a way for the development of specific therapeutic modalities.

## Conclusions

The higher protein levels and mRNA gene expression of CD163 were seen in generalized chronic periodontitis subjects with type II diabetes mellitus. This indicates protein involvement in the progression of the pathogenesis of periodontal inflammation in type II diabetes mellitus patients. Within the limitation, this study projects the CD163 protein as an important biomarker that predicts the outburst, diagnosis, prognosis, and treatment of disease activity.
